# DNA repair in darkness: evolutionary conservation of photolyase function beyond light

**DOI:** 10.1038/s42003-026-09750-4

**Published:** 2026-02-27

**Authors:** Yuhang Hong, Yi Huang, Zhiqiu Huang

**Affiliations:** 1 Key Laboratory of Application of Ecology and Environmental Protection in Plateau Wetland of Sichuan, Xichang University, Xichang, Sichuan Province China; 2 Key Laboratory of Animal Disease Detection and Prevention in Panxi District, Xichang University, Xichang, China

**Keywords:** DNA damage and repair, Molecular evolution

## Abstract

A recent cavefish study overturns the classic view of photolyases as light-dependent enzymes. Research shows CPD photolyase repairs oxidative DNA damage in darkness, revealing a dual function that explains its conservation in cave species

## Teaser

DNA repair by photolyases has classically been viewed as a light-dependent process, restricted to the reversal of UV-induced pyrimidine dimers. A recent study published in Nature Communications overturns this paradigm by showing that CPD photolyase in the blind cavefish *Phreatichthys andruzzii* is still involved in DNA repair function in complete darkness. Using comparative models of surface-dwelling and cave-dwelling fish, the authors demonstrate that CPD photolyase not only mediates photoreactivation under light but also protects against oxidative DNA lesions in the absence of light, explaining its remarkable evolutionary conservation.

**Fig. 1:**
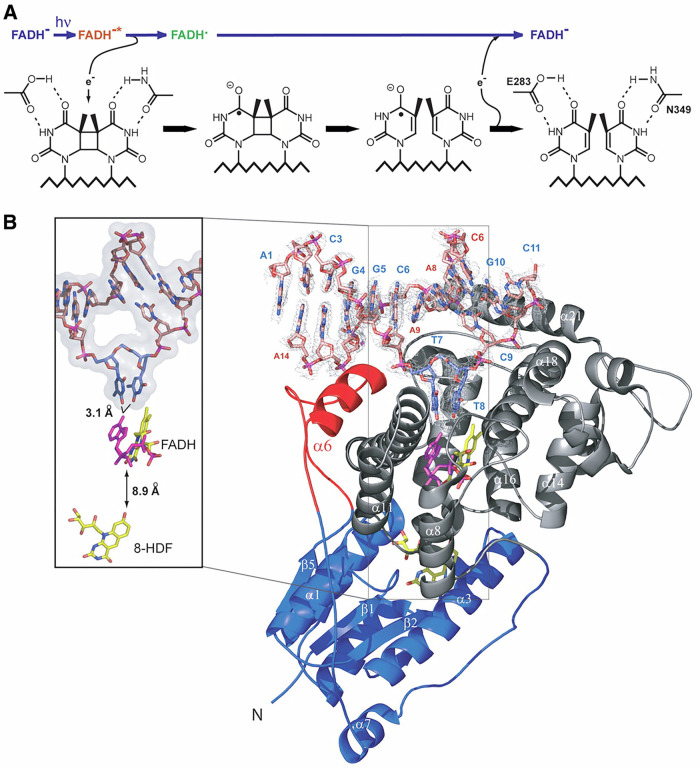
Structural basis of CPD photolyase and the principle of light-driven DNA repair. **A** Schematic of the photoreactivation mechanism. After absorption of visible light, the FADH^-^ cofactor is excited and initiates electron transfer through the conserved catalytic residues (e.g., E283, N349), leading to cleavage and repair of UV-induced cyclobutane pyrimidine dimers (CPDs) in DNA. **B** Crystal structure of CPD photolyase bound to damaged DNA. The DNA duplex is shown with the flipped-out CPD lesion inserted into the active pocket near FADH^-^. The electron density highlights lesion positioning, with the tryptophan electron-transfer chain and antenna cofactor 8-HDF indicated. This structural view illustrates how CPD photolyase recognizes DNA damage and performs ultrafast, light-dependent repair. The use of this figure is authorized by The American Association for the Advancement of Science (License No. 6130220919755)^4^.

Ultraviolet (UV) radiation from sunlight is a well-known driver of DNA damage, producing cyclobutane pyrimidine dimers (CPDs) and (6-4) photoproducts (6-4pps) that threaten genomic stability^1^. Photolyases, enzymes that catalyse light-driven repair of such lesions, are conserved across many taxa and usually considered indispensable in light-exposed environments^2^. CPD photolyases are ancient flavoproteins and can perform remarkably efficient, light-driven reversal of cyclobutane pyrimidine dimers (CPDs) in double-strand DNA, named photoreactivation, to protect genomic DNA integrity^3^. The canonical mechanism that photoreduction of a FAD cofactor followed by rapid electron transfer into the flipped CPD and bond scission has been elucidated in exquisite structural and spectroscopic detail. High-resolution co-crystal structures show how photolyases bind duplex DNA, flip the CPD into a tight catalytic pocket and position cofactors for ultrafast electron transfer; a conserved triad of tryptophans commonly forms an electron transfer chain that relays photoexcitation from a surface antenna chromophore to the active FAD center, enabling lesion repair in picoseconds (Fig. 1)^4^. These structural themes including DNA flipping, FAD chemistry and electron-transfer pathways are conserved across bacterial, archaeal and eukaryotic CPD photolyases, although family members fall into distinct structural classes with nuanced differences in antenna binding and substrate contacts that affect lesion specificity and photochemistry.

Within animals, the presence and architecture of photolyases are taxon-dependent: fishes, amphibians, reptiles and birds typically retain CPD photolyase genes, whereas placental mammals have lost photolyases and rely entirely on nucleotide excision repair (NER) and other pathways to handle UV lesions^5^. Phylogenetic analyses of the cryptochrome/photolyase superfamily (CPF) reveal repeated lineage-specific loss events and domain shuffling, and indicate that eukaryotic photolyases are, on average, under weaker constraint than their bacterial counterparts- but important functional residues (FAD-binding motifs and the Trp triad) remain highly conserved where the gene is retained^6,7^. This mixed picture suggests that selection on photolyases depends heavily on ecological exposure to UV as well as on the capacity of other DNA repair systems to compensate.

Yet organisms inhabiting perpetual darkness, such as cavefish, provide an intriguing model to ask whether photolyase genes are dispensable relics or whether they retain essential functions in the absence of sunlight. In a recent study, Li et al. (2025) investigated the blind cavefish *Phreatichthys andruzzii*, which has been isolated from UV exposure for millions of years, to determine how its photolyase genes have evolved^8^. Their analyses revealed that both 6-4 photolyase and Cry-DASH photolyase genes exhibit truncations, loss-of-function mutations, and extensive polymorphism, suggesting relaxed selection. In contrast, the CPD photolyase gene remains remarkably conserved, showing little evidence of functional decay. This pattern of differential retention points to strong selective constraint maintaining CPD photolyase in the cavefish genome despite the lack of sunlight. Functional experiments strengthened this conclusion. Using CRISPR knockouts in medaka, Li *et al*. demonstrated that loss of CPD photolyase leads to elevated DNA damage levels and reduced survival under oxidative stress, even in complete darkness. Complementary gain-of-function assays showed that expressing zebrafish CPD photolyase in mammalian fibroblasts restored not only light-driven DNA repair but also conferred protection against oxidative stress in the dark. Strikingly, the enzyme’s role extended beyond UV-induced lesion repair: it also mitigated accumulation of oxidative lesions such as 8-hydroxy-deoxyguanosine (8-OHdG). Structural insights further revealed that the canonical tryptophan electron transfer chain, essential for photoreactivation, was also required for this light-independent protective effect, underscoring that the potentially same catalytic machinery supports both modes of action.

**Fig. 2:**
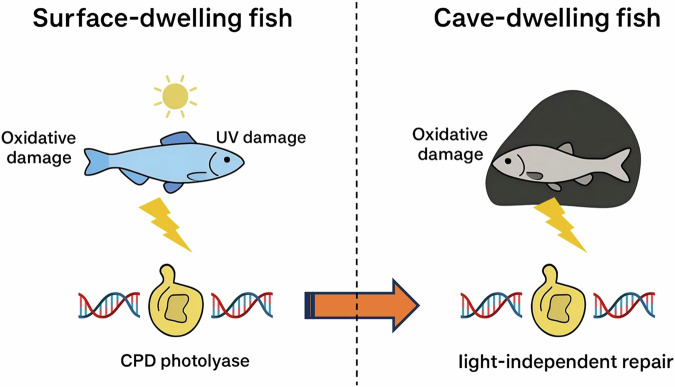
Comparative model of DNA repair functions in surface-dwelling and cave-dwelling fish. In surface fish exposed to sunlight, CPD photolyase not only performs classical photoreactivation of UV-induced CPDs, but also be involved in repairing oxidative lesions. In contrast, the blind cavefish (*Phreatichthys andruzzii*) has evolved in complete darkness for millions of years, where 6-4 photolyase and DASH photolyase genes are pseudogenized, but CPD photolyase is strictly conserved. Experimental evidence demonstrates that CPD photolyase in cavefish protects against oxidative DNA damage (e.g., 8-OHdG) under dark conditions, revealing a light-independent repair capacity. This dual functionality explains the evolutionary persistence of CPD photolyase and provides new insight into DNA repair adaptation to extreme environments. The image and all elements was created by the authors using a WPS Office software (Beijing Kingsoft Office Software, Inc., China).

The discovery that CPD photolyase operates in darkness adds a new dimension to our understanding of DNA repair evolution. Previous work in *P. andruzzii* had already suggested that regulatory networks for photolyase induction by light and reactive oxygen species had degenerated, while nucleotide excision repair components such as ddb2 remained inducible^9^. Broader phylogenetic analyses of the CPF have also shown lineage-specific losses in mammals and other vertebrates, consistent with the idea that photolyase utility varies with environmental light regimes^10^. In microbes and cyanobacteria, the existence of bifunctional photolyases able to act on multiple substrates highlights the evolutionary flexibility of this enzyme family^10^. Against this backdrop, the retention of CPD photolyase in cavefish suggests that its function is not restricted to photoreactivation, but extends to repair of oxidative stress-induced DNA damage (Fig. 2).

The evolutionary significance of this finding lies in the recognition that DNA damage is not driven solely by environmental UV but also by intrinsic metabolic processes. In the perpetual darkness of caves, oxidative stress appears to have provided sufficient selective pressure to conserve CPD photolyase, while other photolyases lacking similar dark activity have been lost. This nuanced pattern of retention and decay reflects the complex interplay between environment, metabolism, and DNA repair needs. The case of cavefish thereby bridges our understanding of how repair enzymes may diversify or persist outside of their canonical ecological niches^11^.

Several open questions remain. The precise molecular mechanism of dark repair is still unclear: whether CPD photolyase directly catalyses lesion reversal in the absence of light or acts as a lesion sensor that recruits other repair systems such as base excision or nucleotide excision repair is yet to be determined. Structural studies capturing photolyase bound to oxidative lesions could clarify how recognition and catalysis occur. Expanding genetic surveys across multiple cave and surface fish populations will also reveal whether the conservation of CPD photolyase is universal or context-dependent. Finally, the cross-species protection observed in mammalian cells raises the possibility of translational applications. Topical formulations containing photolyase extracts have been explored to reduce UV-induced skin damage, and recent work shows that delivery of photolyase coding mRNA or recombinant protein can transiently confer lesion-specific repair activity in mammalian cells that otherwise lack photolyase. If CPD photolyases can materially reduce oxidative as well as UV lesions in mammalian contexts, engineered or delivered photolyases might complement NER/BER in high-risk tissues, for example, in photodamaged skin, in organs exposed to oxidative inflammation, or in ex vivo gene-therapy settings to lessen mutational burden. Any clinical translation would require careful evaluation of off-target chemistry, redox perturbations, immune responses to foreign proteins, and long-term genomic stability. Nevertheless, the possibility that an ancient enzyme can be repurposed to mitigate modern human pathologies is provocative and warrants rigorous biochemical and safety studies.

In summary, Li et al.’s work thus compels a reassessment of photolyase biology. Far from being a redundant enzyme in the absence of light, CPD photolyase emerges as a guardian of genome integrity in darkness. By uncovering this dual functionality, the study highlights the need to consider both environmental and metabolic sources of DNA damage when interpreting the evolution of repair systems. More broadly, it suggests that other DNA repair enzymes may harbour unappreciated roles under extreme or atypical conditions, opening new avenues for both evolutionary biology and biomedical research.
